# The need for post-stroke cognitive screening - the rationale behind the Hungarian adaptation of the Oxford Cognitive Screen (OCS) and its pilot study

**DOI:** 10.1016/j.cccb.2025.100527

**Published:** 2025-12-18

**Authors:** Tímea Tünde Takács, Judit Kárpáti, Edina Szabó, Károlyné Pálvölgyi, Panna Pálinkás, Orsolya Antal, Júlia Baross, Bernadett Bruckner, Sam Webb, Nele Demeyere, Bence Gunda

**Affiliations:** a Semmelweis University, Department of Neurology, Budapest, Hungary; b Semmelweis University, Faculty of Health Sciences, Department of Voice, Speech and Swallowing Therapy, Budapest, Hungary; c Semmelweis University, Faculty of Medicine, Budapest, Hungary; d Bajcsy-Zsilinszky Hospital and Clinic, Department of Musculoskeletal Rehabilitation, Budapest, Hungary; e Doctoral School of Psychology, ELTE Eötvös Loránd University, Budapest, Hungary; f Institute of Psychology, ELTE Eötvös Loránd University, Budapest, Hungary; g Department of Neurology, Bajcsy-Zsilinszky Hospital, Budapest, Hungary; h Nuffield Department of Clinical Neurosciences, University of Oxford, Oxford, United Kingdom

**Keywords:** Post-stroke cognitive impairment, Stroke protocol, Hungary, Oxford cognitive screen, patient safety

## Abstract

**Background:**

Post-stroke cognitive impairment (PSCI) is a frequent yet underdiagnosed consequence of stroke, with significant implications for quality of life, functional outcomes and long-term prognosis. Despite its importance, cognitive screening remains insufficiently integrated into post-stroke care protocols, particularly in Hungary.

**Objective:**

This study aimed to adapt and pilot the Oxford Cognitive Screen (OCS) for Hungarian stroke patients, addressing the urgent need for a culturally and linguistically appropriate, stroke-specific cognitive screening tool that is freely available in Hungarian.

**Methods:**

The Hungarian adaptation followed a rigorous 11-step linguistic validation process, including cultural adaptation, forward and back translations, reviews, and pilot testing. Ten stroke patients at Semmelweis University were assessed using the adapted OCS within three weeks of symptom onset. Data on test performance, feasibility, and patient feedback were collected from 10 pilot patients and 6 examiners.

**Results:**

The adaptation and pilot testing process, completed in approximately three months, confirmed the usability of the tool, with most patients finding it engaging. The median number of impaired tasks was two. No major issues arose, and the tool was well received by both patients and examiners.

**Conclusion:**

The Hungarian version of the OCS offers a practical, user-friendly cognitive screening tool tailored for stroke patients. Its implementation could enhance early detection of PSCI, improve patient safety by supporting targeted rehabilitation, and ultimately contribute to better long-term outcomes. This initiative represents a foundational step toward integrating cognitive screening into stroke protocols in Hungary, addressing a significant gap in rehabilitation and healthcare equity.

## Highlights

• First Hungarian adaptation of a stroke-specific neuropsychological screening tool, the Oxford Cognitive Screen.
• Provides a culturally and linguistically validated, stroke-specific tool.
• Pilot study confirmed feasibility and strong acceptance by patients and clinicians.
• Fills a critical gap in stroke care by facilitating cognitive screening in Hungary.
• Supports earlier detection of PSCI, guiding rehabilitation and improving outcomes.

## Introduction

Stroke is a common, leading, and growing cause of disability worldwide [1,2]. Its physical, psychological and financial impacts place a heavy burden on healthcare systems and society [1]. According to 2021 data, the global stroke burden exceeds $721 billion annually, and stroke is the leading and growing cause of disability-adjusted life years (DALY) [3]. Following a stroke, about 50 % of patients experience long-term neurological symptoms [4], and only about 40–60 % of young patients are able to return to work, representing a significant loss of productivity [5,6].

While physical stroke symptoms (central facial palsy, hemiparesis, sensory dysfunction, disturbances of speech and language, disorders of the visual field and vertigo) are relatively easy to recognize and result in a prompt presentation, cognitive symptoms are often neglected. Recent advancements in stroke care focus on the accessibility of acute reperfusion therapies; however, the percentage of patients receiving such treatments remains low, at approximately 7–10 % for intravenous thrombolysis (IVT) and 2–5 % for endovascular therapy (EVT), with significant intra- and inter-country differences [7]. Despite its high prevalence, post-stroke cognitive impairment (PSCI) remains underdiagnosed, under-researched and inadequately addressed in diagnostic and treatment protocols [8,9]. Initial studies estimated the prevalence of cognitive impairment in approximately one-third of patients [10], while more recent studies have shown that 50–83 % of stroke survivors [[11], [12], [13], [14]] have persistent neurocognitive symptoms. Notably, 40–71 % of neurologically asymptomatic or nearly asymptomatic patients were found to have cognitive deficits affecting at least one domain [13,[15], [16], [17]]. Even temporary cognitive impairment after a stroke increases the likelihood of developing dementia later [18]. Approximately one-third of patients with transient ischemic attacks (TIA) exhibit cognitive impairment [19,20]. A study showed that the incidence of post-event dementia at 1 year increases with increasing stroke severity among other factors, highlighting a strong correlation between neurological impairment and early cognitive decline [21].

Cognitive impairment in various domains showed a significant correlation with the quality of life of patients and caregivers [22], the long-term functional outcomes [13], the risk of recurrent stroke, future dementia [20,21], and mortality of patients [23,24]. Cognitive screening is crucial as patient-perceived cognitive impairments such as attention, memory, and speech difficulties are the most common unmet needs [25] and often align with objectively measured multi-domain, cumulative cognitive dysfunction [26]. The benefit of cognitive training most pronounced within the first 6–12 months after a stroke, with only limited gains observed beyond this period. Thus, early detection and intervention are critical to achieving the best cognitive recovery outcomes. Undetected cognitive impairment poses significant patient safety risks, including an increased likelihood of falls [27], potential medication errors [28], and misinterpretation of medical instructions.

The availability of cognitive screening tools in Hungarian is limited, and they focus on the detection of Alzheimer's Disease (Mini Mental State Exam (MMSE) [29], Montreal Cognitive Assessment (MoCA) [30], Test Your Memory [31], Alzheimer's Disease Assessment Scale - Cognitive subscale [32] and the 7-Minute-Test [33]). None of these were tested or validated in Hungarian solely on a post-stroke patient cohort. The healthcare and personal burden of PSCI underscores the necessity of implementing a stroke-specific cognitive screening tool. Such a tool should be (i) easy to administer, not only by neuropsychologists but also other healthcare professionals; (ii) stroke-specific, with domain-targeted specific cognitive tests that reflect the cognitive profile of stroke, which differs from dementia profiling, (iii) quick, (iv) free of charge, and (v) available for both initial and follow-up examinations. The Oxford Cognitive Screen (OCS), which was originally tested on 140 healthy and 208 stroke patients, showed an overall sensitivity of 87.7 % compared to MoCA [34] and 100 % compared to MMSE [35], with good score correlation between the OCS and MoCA over time [36]. The OCS contains 10 subtests for 5 different cognitive domains: attention, language, number, praxis, and memory, resulting in a total of 14 different scores. The OCS, being more inclusive than MoCA and MMSE for patients with aphasia and neglect syndrome, fulfills all the criteria mentioned above [37]. Its stroke-specific design, domain-based rather than overall score interpretation, and brief administration time (15–25 mins) make it suitable for post-stroke cognitive screening. However, its limited sensitivity to memory deficits, potential confounding from non-dominant hand use, and overlap in domain sampling may reduce its precision in detecting subtle or isolated impairments [38]. As the OCS is a comprehensive, stroke-specific cognitive screening tool with a growing number of linguistic validations [39], we found it worthwhile to undertake its linguistic validation and make it available in Hungarian.

## Methods

The Hungarian adaptation of the OCS was carried out following approval by Oxford University Innovation Limited (license number 00OCS-1005,800), based on their provided Translation and Linguistic Validation Process and Concept Elaboration Report. The order of tasks was as follows:

1. Cultural adaptation of stimuli/tasks: All images, words, and sentences were adapted to ensure cultural appropriateness and familiarity for the target population, while maintaining the conceptual integrity of the original test, as indicated in the Concept Elaboration Report.
2. Cultural adaptation Reconciliation: Proposed cultural adaptation ideas were submitted to the OCS developers for review and approval to ensure that new stimuli or wording align with the original materials and rationale.
3. Forward translation: Two independent translators separately prepared translations of the OCS into the target language, following the source documents and the concept elaboration guidelines.
4. Forward translation review: The project manager, translators and OCS developers reviewed the forward translations to confirm consistency, formatting accuracy, and adherence to the original test layout.
5. Back-translation: The reconciled translation was independently back-translated by into English by two translators who have not seen the original OCS in English.
6. Back translation review: The project manager, translators and OCS developers reviewed the back-translated version against the original to identify and resolve any discrepancies in meaning or terminology.
7. Pilot testing: The translated version was administered to a small group of native speakers to evaluate clarity, comprehension, and cultural relevance through direct feedback.
8. Pilot testing review: The project manager reviewed feedback from pilot testing and coordinated any necessary revisions to enhance accuracy and participant understanding.
9. Proofreading: The final translation underwent two rounds of proofreading by independent reviewers to correct linguistic and formatting errors before approval.
10. Normative Data Collection A sample of at least 60 native speakers will complete the translated OCS to establish appropriate culturally and linguistically appropriate cutoff scores for impairment- yet to be completed.
11. Publication of normative and psychometric data of the target language - yet to be completed.

Our team consisted of a neuropsychologist, a linguist and speech therapist, a chief nurse, two neurologists, and translators with no healthcare background. We analyzed the original tests and other materials to identify items that required cultural adaptation. The most challenging aspect was the adaptation of the sentence according to the Concept Elaboration Report. Hungarian is an agglutinative language, whereas English is isolating-inflectional. Agglutinative languages express grammatical relations with suffixes attached to the word stem, resulting in longer words with more morphemes.

Therefore, in consultation with the original authors, we decided to shorten the Hungarian sentences to ten words to match the English morpheme count. As the length of the sentence changed, we used a maximum of 10 points for scoring instead of the 15 points for 15 words, and set the cut-off for impairment at 9 instead of 14 in the source. This scoring will be later used during the normative data collection phase, which will be the topic of a further paper. This change did not affect the delayed recall and recognition tasks, nor their scoring, it only altered the word options in these tasks.

There was some debate about changing the filing cabinet picture, as it is not commonly used in Hungary. However, we ultimately decided to adhere as closely as possible to the original test and therefore we kept it. We translated the patient packs, test booklets, and user manual according to the provided instructions. For both forward and backward translations, two unbiased translators had access to the source material and the Concept Elaboration Report. During the translation review, the team discussed the differences between translations and selected the version that best aligned with the Concept Elaboration Report.

Pilot testing was conducted in accordance with the instructions outlined in the Translation and Linguistic Validation Process: A minimum of five native Hungarian participants completed the translated version of the OCS. During individual face-to-face sessions, participants were asked to comment on the response options in the translated version, identify any wording that was difficult to understand, suggest alternative phrasing where necessary, and confirm that the translation was understandable. We tested ten patients with imaging-confirmed stroke at Semmelweis University Department of Neurology within three weeks of the stroke event. The findings from the pilot testing review are presented in this article. The collection of normative data is currently underway to determine the language-specific cutoffs and finalize the validation process. These results will be reported in a future publication.

## Results

The Hungarian adaptation of the OCS was completed over approximately three months. Pilot testing was conducted over a two-week period with ten patients diagnosed with acute ischemic stroke within three weeks from symptom onset.

The mean (SD) age of the sample was 65.8 (11.9) years; seven participants (70 %) were male. Their mean duration of education was 10.8 (2.1) years (corresponding to secondary vocational education). All participants had an ischemic stroke, two in the posterior fossa, two with multiple lesions and the remaining six in the left hemisphere. The median NIHSS was 3 (range 2–11), and the median modified Rankin Scale score was 2 (range 1–4). Testing was performed on average 5.7 days (range 2–10) after symptom onset. Seven assessments were performed at the bedside and three in an office setting.

Overall, patient feedback was positive. Participants reported that the instructions were clear and that the test was understandable and acceptable. Minor issues arose in two specific subtests: 1. Broken Hearts: two participants reported that the hearts tended to blur after prolonged observation. 2. Praxis: Three participants required additional instructions - particularly regarding hand positioning and facing - to complete the praxis test. The sentence-reading task included less familiar Hungarian words, which some participants found amusing but still comprehensible. No changes to wording were requested.

The scoring required some practice on the part of the examiners. We made sure to have a varied examiner group in our pilot: two tests were performed by a neurologist, two by a chief nurse, and three by a research student and a speech therapist. The test results showed a median of 2 affected tasks, ranging from 0 to 8. Scoring was generally straightforward and consistent across examiners. The praxis subtest required occasional consultation of the user manual to ensure scoring accuracy. Examiners observed a brief learning curve when managing test materials and scoring simultaneously, which improved with practice.

The pilot primarily aimed to evaluate the linguistic and procedural feasibility of the translated OCS rather than its psychometric validity; therefore, no formal comparison was made between the Hungarian OCS and other cognitive screening tools. Nevertheless, all participants were able to complete the full OCS battery within 15–20 min.

Overall, the pilot study of the Hungarian OCS was found to be feasible, acceptable, and easily comprehensible for acute stroke patients. No major linguistic or cultural adaptations were required following pilot testing. The pilot provided valuable experience in administration and scoring, which will contribute to future validation.

## Discussion

This pilot study represents the first step toward the use of OCS with Hungarian-speaking stroke patients. The results indicate that the Hungarian version of the OCS is linguistically clear, culturally appropriate, and feasible to administerin an acute stroke setting, thus its later validation will make it a practical tool for the everyday clinical practice.

Although PSCI is one of the most common sequalae of stroke, cognitive screening is still not systematically integrated into stroke care. The main barrier is typically not the condition of the patient but rather the lack of system-level integration and standardized protocols. Therefore, developing and adhering to local protocols for this purpose is imminent [40]. The World Health Organization (WHO) has recognized the importance of rehabilitation and aims to emphasize the development and strengthening of rehabilitation at all levels in all countries by 2030 [41]. Internationally, access to rehabilitation varies between 13–57 % relative to the actual need [42]. Cognitive rehabilitation is particularly limited in many countries [43], and patients are often unaware of these opportunities [44].

In Hungary, rehabilitation capacity has declined in recent years, with the number of rehabilitation beds decreasing from 14,707 in 2019 to 13,134 in 2023 (for both neurological and non-neurological patients). The annual incidence of stroke in Hungary is estimated at 30,000–40,000 cases, although precise data are lacking due to the lack of a national registry. Empirical research suggests that approximately one-third of these patients would require institutional rehabilitation [45], which means that limited rehabilitation availability already constrains post-stroke care. Another significant issue is the tendency of cherry picking of stroke patients with better social backgrounds and milder strokes for rehabilitation, as opposed to those with more severe strokes from lower social status groups, who may have undergone extensive diagnostic and treatment efforts during the acute phase.

Furthermore, post-discharge care for patients returning home from an acute ward - whether after institutional rehabilitation or not - remains heavily dependent on the proactivity of the general practitioner and their social network. In Hungary, functional improvement is not a primary indicator [45] for primary care physicians and the availability of specialized care such as physiotherapy, speech therapy and neuropsychology services near the patient's residence is very limited. The patient's social status, along with willingness, proactivity and persistence of their environment to provide support, plays a crucial role in post-stroke recovery. The general shortage of healthcare and social professionals is only partially alleviated by enhancing rehabilitation training and raising awareness in primary care [46]; therefore, a coordinated national stroke care pathway strategy, including cognitive screening, is required,.

There is no universally accepted standard for post-stroke cognitive screening: some authors suggest individualized neuropsychological examination for those with multiple risk factors [47], while recent manuscripts and the scientific guidelines of the American Heart and Stroke Association recommend systematic cognitive screening according to local possibilities [[48], [49], [50]]. Although current guidelines do not recommend routine, systematic neuropsychological screening for stroke patients due to the lack of evidence in large-scale, population-level studies [51], the impact of cognitive performance on rehabilitation, functional outcomes, and thus the social and economic burden of stroke is becoming increasingly apparent. Accordingly, both European and American guidelines recommend post-stroke cognitive rehabilitation [[50], [51], [52]].

Moreover, despite these recommendations, there is no ideal screening tool for PSCI. The MMSE and the MoCA are the most commonly used [[53], [54], [55]], which are also available in Hungarian. Of these two dementia screening tests, the MoCA proved to be more suitable in previous studies [56], demonstrating high sensitivity but lower specificity [57]; however, it remains less appropriate for assessing cognitive function in stroke patients [58]. They often underestimate post-stroke cognitive impairment; in the original study, impairment was detected in only 44.1 % of the patient population, compared to 69.3 % when measured with detailed neuropsychological tests [59]. Other studies have found that these tests overestimate cognitive impairment [60,61], mainly due to reduced performance caused by aphasia. Other short tests assess only one cognitive domain (e.g., trail making test, 10-word-list-recall, dual task cost), and their availability in Hungarian is also limited.

Screening tools developed specifically for stroke patients, such as the Birmingham Cognitive Screen (BCoS), the Cognitive Assessment for Stroke Patients (CASP), the Brief Neuropsychological Screening (BNS), the Mild Vascular Cognitive Impairment assessment (MVCI), the Brief Memory and Executive Test (BMET), which is a mild vascular cognitive impairment assessment, and the Northwick Park Examination of Cognition (NPEC), have been validated in smaller cohorts and are not yet widely available or adapted across languages [54]. The BCoS and CASP are more commonly used in research settings. Among these, the OCS has many advantages, and although there is no extensive comparison with other tests, several studies mention it as a promising tool [38,55]. In summary, the psychometric properties of the most widely used MMSE and MoCA tests were not developed for stroke patients; therefore, their usability in this group has been widely criticized. Other tests developed for stroke patients have been validated in smaller patient groups but are not yet usable in diverse linguistic and cultural environments. Therefore, up to this point, no comprehensive PSCI screening test has been available in Hungarian that is quick to administer, easy to teach, and applicable in the acute ward setting. With the soon-to-be-available Hungarian OCS, we hope to fill this gap and offer an appropriate screening tool for post-stroke patients.Our pilot showed that only minor logistical adjustments and brief examiner familiarization are needed to ensure smooth administration. The introduction of the Hungarian OCS will support the early detection of PSCI, thereby guiding tailored rehabilitation interventions and improving long-term functional outcomes.

We consider this initiative as a foundational step toward integrating cognitive screening into local stroke protocols. Our goal is to drive changes in healthcare practices and promote advancements in patient pathways, human resource management, and comprehensive post-stroke care with a focus on complex rehabilitation. There is a timely need to shift the focus from a rather quantitative assessment based on functional outcomes to a more qualitative assessment that considers the quality of life for both patients and caregivers. This qualitative dimension is largely shaped by cognitive functioning.

In our study, the sample size was small (*n* = 10) and homogeneous, which limited the generalizability of the findings. All participants had experienced mild to moderate ischemic stroke. The study design was descriptive, focusing on linguistic and procedural feasibility rather than psychometric validation. Consequently, no statistical analysis of reliability, validity, or sensitivity was performed. For this reason, the pilot did not include a direct comparison with other established cognitive screening tools. Although feedback from both patients and examiners was systematically collected, it was qualitative, which may have introduced response bias. Finally, the pilot was conducted in one acute stroke ward and feasibility may vary in other clinical contexts, such as community or rehabilitation settings. Despite these limitations, this pilot provided essential insights into the linguistic clarity, cultural relevance, and practicality of administration of the Hungarian OCS, establishing an important foundation for future large-scale validation and implementation efforts.

## Conclusions

PSCI is a frequent yet underdiagnosed complication of stroke. Greater emphasis should be placed on cognitive testing and its integration into local protocols. We completed the Hungarian adaptation of the OCS to make a stroke-specific cognitive screening tool available in Hungary.

Recognizing subtle, cognitive impairments justifies targeted rehabilitation, which can improve the quality of patient care. Patients may benefit from more complex rehabilitation, which is vital for long-term social reintegration, management of comorbidities, and the prevention or mitigation of mood disorders and dementia. These impacts are significant from social, economic, and healthcare policy perspectives. Our future goal will be the psychometric validation of the Hungarian OCS. Beyond this, future research may explore how OCS results relate to long-term cognitive and functional outcomes, rehabilitation efficiency, and quality of life. Additional studies could also investigate the feasibility of integrating the OCS into routine clinical workflows and national stroke protocols.

## Funding

This research received no specific grant from any funding agency in the public, commercial, or not-for-profit sectors.

The study was approved by the Ethics Committee of Medical Research Council (approval number: BM/29,860–1/2024).

## CRediT authorship contribution statement

**Tímea Tünde Takács:** Writing – review & editing, Writing – original draft, Project administration, Methodology, Investigation, Data curation, Conceptualization. **Judit Kárpáti:** Supervision. **Edina Szabó:** Supervision, Investigation, Conceptualization. **Károlyné Pálvölgyi:** Data curation. **Panna Pálinkás:** Investigation, Data curation. **Orsolya Antal:** Investigation. **Júlia Baross:** Investigation, Data curation. **Bernadett Bruckner:** Investigation. **Sam Webb:** Formal analysis, Data curation. **Nele Demeyere:** Validation, Supervision, Resources, Methodology, Conceptualization. **Bence Gunda:** Writing – review & editing, Validation, Supervision, Conceptualization.

## Declaration of competing interest

The authors declare that they have no known competing financial interests or personal relationships that could have appeared to influence the work reported in this paper.
